# A highly porous and conductive composite gate electrode for OTFT sensors†

**DOI:** 10.1039/c9ra00148d

**Published:** 2019-03-04

**Authors:** Soniya D. Yambem, Samantha Burns, Joshua N. Arthur, Jana Timm, Maria A. Woodruff, Ajay K. Pandey, Roland Marschall

**Affiliations:** School of Chemistry Physics and Mechanical Engineering, Science and Engineering Faculty, Queensland University of Technology (QUT) Brisbane QLD 4000 Australia soniya.yambem@qut.edu.au; Institute of Health and Biomedical Innovation, Queensland University of Technology (QUT) Kelvin Grove Queensland 4059 Australia; Department of Chemistry, University of Bayreuth Bayreuth 95447 Germany; School of Electrical Engineering and Computer Science, Science and Engineering Faculty, Queensland University of Technology (QUT) Brisbane QLD 4000 Australia

## Abstract

Ionic/protonic to electronic transducers based on organic thin film transistors have shown great promise for applications in bioelectronic interface devices and biosensors, and development of materials that exhibit mixed ionic/electronic conduction are an essential part of these devices. In this work, we investigated the proton sensing properties of an all solid-state and low voltage operating organic thin film transistor (OTFT) that uses the organic mixed conductor poly(3,4-ethylenedioxythiophene) doped with poly(styrene sulfonate) (PEDOT:PSS) as the gate electrode. To address the limited sensitivity due to the lack of porosity in PEDOT:PSS base sensors, we proposed a composite gate electrode material composed of PEDOT:PSS and proton conducting mesoporous SO_3_H-Si-MCM-41 nanoparticles for improved proton sensitivity. The composite gate electrode doubles the proton sensitivity of the OTFT, indicating a clear advantage of adding SO_3_H-Si-MCM-41 in the PEDOT:PSS gate. Moreover, the OTFTs with the composite gate electrode maintained OTFT characteristics similar to that of the PEDOT:PSS gated OTFT. A detailed and systematic study of the effect of variation in the composition of PEDOT:PSS:SO_3_H-Si-MCM-41 on OTFT characteristics and sensing properties is carried out. Our results open up the possibility of combining inorganic nanomaterials with organic conductors in the development of highly efficient bioelectronic sensing platforms.

## Abstract 2

A porous and conductive gate electrode of PEDOT:PSS and sulphonated mesoporous silica nanoparticles is investigated for organic thin film transistor sensors.

## Graphical abstract

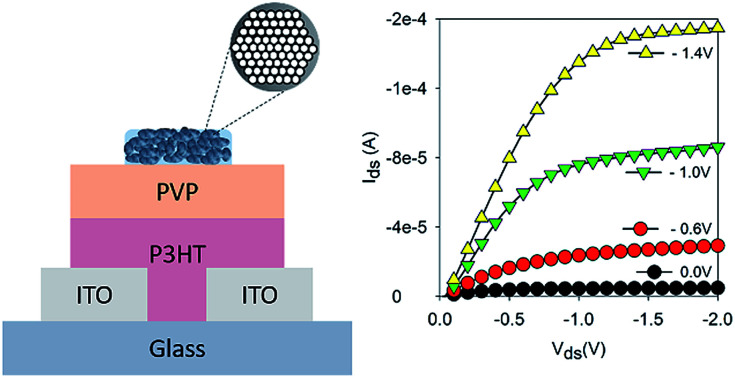

## 1. Introduction

Ion sensitive electronic devices are highly desirable for developing biosensors and bioelectronic interface devices for incorporation in smart prosthetics that can communicate with the user. In particular, ion sensitive devices based on organic electronics are very attractive since organic electronic devices have desirable attributes such as flexibility, stretchability, lightweight properties and printability.^1–4^ These attributes are essential for developing devices with complex functionalities, which can be integrated with soft tissues in biological systems. Among organic electronic devices, organic electrochemical transistors (OECTs) are one of the most widely investigated devices for bioelectronics and biosensors.^5^ OECTs have low operating voltages and are efficient transducers of ionic signals to electronic signals. As such, OECTs have been successfully demonstrated for many bioelectronic interface devices, for example, recordings of brain activity and neuron simulation.^6,7^

Whilst OECTs have shown great promise, it is desirable to have an all solid-state device to circumvent issues and challenges related to containment of the electrolyte in the device. In an OECT, an electrolyte is in direct contact with the organic semiconductor film. All solid-state biosensors based on organic thin film transistors (OTFTs) have been achieved, where the gate electrode is a polymer with ionic properties and the redox enzyme is immobilized on top or in the gate electrode.^8^ This success of all solid-state biosensor indicates that mixed (ionic/electronic) conductors have great potential for incorporation as gate electrodes in OTFTs for ion sensing. Among mixed conductors, the most common material is poly(3,4-ethylenedioxythiophene) doped with poly(styrene sulfonate); (PEDOT:PSS) and is widely used in OECTs.^9^ Additionally, PEDOT:PSS gated OTFTs have been shown to operate at low voltages, 0 > *V*_ds_ > −2 V.^10,11^ However, due to limited porosity of PEDOT:PSS films, ion sensing properties of PEDOT:PSS gated OTFTs are yet to be realised.

In this work, we investigate the proton sensitivity of an all solid-state and low voltage operating OTFT with PEDOT:PSS as the gate. The proton sensitivity of the OTFT was further enhanced by adding proton conducting mesoporous SO_3_H-Si-MCM-41 nanoparticles in the PEDOT:PSS gate and forming a composite gate electrode.^12^ We show that the OTFTs with composite gate electrodes are particularly beneficial in increasing the proton sensitivity. Our findings are significant and pave the way for development of all solid-state, low voltage operating ionic to electronic transducers, which are highly applicable in bioelectronics and biosensors.

## 2. Experimental methods

### 2.1 Synthesis & device fabrication

The SO_3_H-Si-MCM-41 nanoparticles (referred to as SNP hereafter) were prepared using a method reported earlier, with 20% co-condensation (molar ratio of tetraethyl orthosilicate to 3-mercaptopropyl trimethoxysilane 80 : 20 for the synthesis).^12^

Pre-patterned indium tin oxide (ITO) glass slides (Xin Yan Technology LTD.) with a 3 mm channel width and 50 μm channel length were used for fabricating OTFTs in this study. The ITO slides were cleaned with Alconox solution in deionized water, followed by consecutive ultrasonication in water, acetone and isopropanol for 10 minutes each. After ultrasonication, the glass slides were blow dried with compressed air. The cleaned ITO slides were spin coated with a 10 mg ml^−1^ poly(3-hexylthiophene-2,5-diyl) (P3HT) in chlorobenzene at 4000 rpm for 30 seconds to give a ∼50 nm thick film. The films were annealed at 60 °C for 10 minutes to remove residual solvent. A poly(4-vinylphenol) (PVP) film was spin coated on top of the P3HT film from an 80 mg ml^−1^ solution in ethanol at 4000 rpm for 60 seconds to give a ∼500 nm thick film. This was followed by annealing at 85 °C for 20 min. After each spin coating step and before annealing, the films were patterned to ensure the films between adjacent devices were not connected as this could lead to large leakage current and hence non-optimal or non-working devices. For PEDOT:PSS gated OTFTs, 10 μl of PEDOT:PSS (Heraeus, CLEVIOS™ P VP AI 4083) was drop-casted on top of the channel area. For OTFTs with 100% SNP gate, a 10 μl drop of 10 mg ml^−1^ SNP suspension in de-ionised water was used. The suspensions were prepared by stirring the nanoparticles in de-ionised water untill it formed a homogeneous suspension. For PEDOT:PSS : SNP composite gate electrodes, PEDOT:PSS and SNP suspension were mixed in desired ratios (v/v) and stirred on a magnetic stirring plate to form a homogeneous solution. 10 μl of this composite solution was used to make the gate electrode. The gate electrodes for all OTFTs were defined using a scotch tape mask to ensure the same dimensions. All gate electrodes were dried at room temperature. For *IV* measurements of the gate electrodes, the gate materials with same dimension were deposited on ITO contact pads.

### 2.2 Characterization & testing

All OTFTs were tested immediately after fabrication. Output and transfer characteristics were measured using a Keysight B1500A semiconductor analyser. For proton sensing measurements, 2.5 μl of H_2_O_2_ (30 wt% in H_2_O, Sigma Aldrich) was drop-casted on top of the gate electrode while the OTFT was operating at *V*_ds_ = *V*_g_ = −1 V and drain current with respect to time were recorded. *IV* curves of the gate electrode materials were also taken using the Keysight B1500A semiconductor analyser. Thicknesses of the constituent thin films were measured using a Dektak profilometer. The images of the gate electrodes were taken using a Zeiss Orion Helium Ion Microscope.

All fabrication and testing of the OTFTs reported were done in ambient environment.

## 3. Results & discussion

**Fig. 1:**
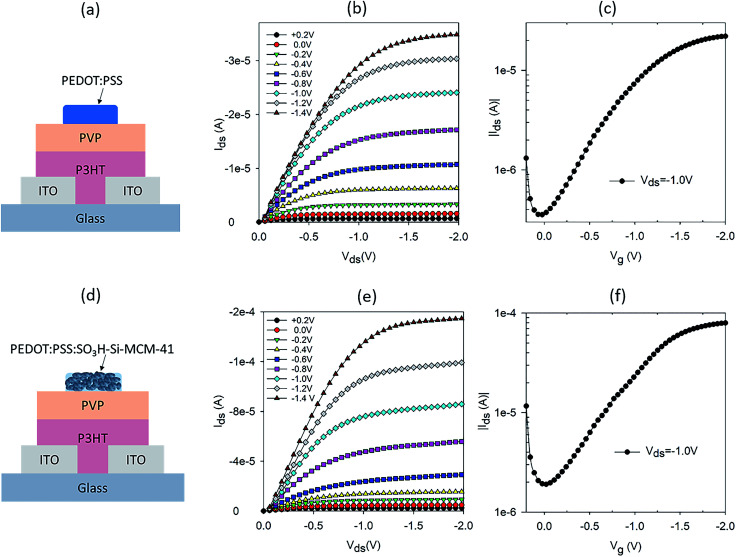
(a) Schematic of a PEDOT:PSS gated OTFT and its (b) output and (c) transfer characteristics. (d) Schematic of OTFT with a composite gate electrode of PEDOT:PSS and SNP (50 : 50, v/v) and its (e) output and (f) transfer characteristics.

OTFTs with low operating voltages have been demonstrated using high-*k* dielectric materials in bottom gate top contact structures.^13–15^ In top gate bottom contact configurations, OTFTs with a hygroscopic dielectric layer have been shown to operate at low voltages.^10,11^ For our study, a top gate OTFT configuration was chosen to allow the ion (or proton) sensitive gate to directly interact with the analyte or source of ions. A schematic of the OTFT structure is shown in Fig. 1a, where PVP was chosen as the hygroscopic dielectric layer. P3HT is used as the channel material and ITO as the source and drain electrodes. A readily available commercial PEDOT:PSS was used as the gate electrode.

Apart from the source and drain electrodes, all materials used in our OTFT are solution processable. The output and transfer characteristics of the PEDOT:PSS gated OTFT are shown in Fig. 1b and c, respectively. Well defined linear and saturation regions are seen for operating voltages 0 > *V*_ds_ > −2 V. This operating voltage regime is typical of OTFT with a hygroscopic dielectric layer.^10^ The transfer characteristic in Fig. 1c, shows that the ON/OFF ratio is not very high. However, lower ON/OFF ratios are typical of OTFTs with a hygroscopic dielectric layer.^10^ To test the proton sensitivity of the OTFT, we dropped 2.5 μl of hydrogen peroxide (H_2_O_2_) on the gate electrode, while the OTFT was in operation (*V*_ds_ = −1 V and *V*_g_ = −1 V) and monitored the current between source and drain, *I*_ds_, with time. The operating condition chosen is appropriate for electrochemical breakdown of H_2_O_2_ into protons, electrons and water.^8^ With a gate that has ionic properties, the protons diffuse through to the dielectric layer and resulted in doping of the semiconducting channel.^8,16^ This resulted in a change in the conductivity of the semiconducting layer and hence a modulation in the current between the source and drain. A typical normalised *I*_ds_ response curve for the PEDOT:PSS gated OTFT is shown in Fig. S1a.† The response curve is normalized by the base current before dropping or addition of H_2_O_2_, to account for variation in base current, which is dependent on the initial conductivity of the P3HT film. H_2_O_2_ was added at ∼100 seconds and *I*_ds_ increased immediately, reaching a maximum modulation after 50 seconds of H_2_O_2_ on the gate. The modulation in *I*_ds_ is double of the base current of the OTFT.

To further increase the proton sensitivity of the PEDOT:PSS film, we mixed PEDOT:PSS with mesoporous SNP to form a composite electrode. SNP is one of the best solid-state proton conducting materials.^12^ The SNP used in the composite gate had a pore size of ∼3 nm with a high loading of SO_3_H both inside and outside the pore. In our earlier report, we demonstrated high proton sensitive OTFTs using just SNP as the gate electrode.^17^ However, the transistor characteristics of SNP gated OTFTs were not ideal and had high OFF current and less well defined saturation regions as compared to OTFTs gated with PEDOT:PSS. Nevertheless, results on high proton sensitivity of the SNP gated OTFTs indicates that incorporation of SNP in a good mixed conductor to form a composite gate electrode could be a good strategy to achieve high proton sensitivity. A schematic of the OTFT with composite gate electrode of PEDOT:PSS and SNP is shown in Fig. 1d. The composite gate is a 50 : 50 (v/v) of PEDOT:PSS and 10 mg ml^−1^ SNP in water. Output and transfer characteristics of the OTFTs with composite gate electrode are shown in Fig. 1e and f, respectively. The output characteristics have very well defined linear and saturation regions, similar to that of a PEDOT:PSS gated OTFT (Fig. 1b). The transfer characteristics of the composite gate OTFT and PEDOT:PSS gated OTFT are also similar (Fig. 1f and c). This shows that in spite of 50% of the gate electrode being SNP, the composite gate electrode has good electrical conductivity and is an effective gate for the OTFT. As intended, the proton sensitivity of the composite gate electrode is also higher than a PEDOT:PSS gated OTFT (Fig. S1b†) and a three-fold modulation in *I*_ds_ is achieved for the composite gate electrode as compared to a twofold modulation for a PEDOT:PSS gated OTFT.

**Fig. 2:**
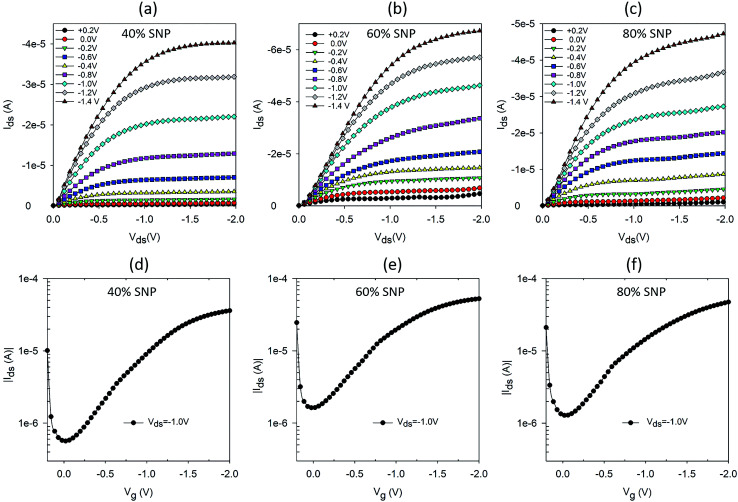
Output and transfer characteristics of OTFTs with PEDOT:PSS : SNP composite gate electrodes with (a and d) 40% (b and e) 60% and (c and f) 80% SNP (v/v).

Following the promising result of achieving higher proton sensitivity with a composite gate electrode of PEDOT:PSS and SNP, we carried out a systematic variation in percentage of SNP in the PEDOT:PSS gate and studied the OTFT characteristics and their proton sensing properties. Five sets of OTFTs with PEDOT:PSS : SNP gates were fabricated. The first set of OTFT had only PEDOT:PSS (0% SNP) and the last set had only SNP (100% SNP) in the gate. The remaining three sets had 40%, 60% and 80% (v/v) SNP in the gate. Output and transfer characteristics of OTFTs with 0% and 100% SNP in gate electrode are provided in Fig. S2.† As expected and similar to our earlier report, the OTFT with 100% SNP had high OFF current (Fig. S2c†) and low amplification (Fig. S2d†).^17^ Output and transfer characteristics of OTFTs with 40%, 60% and 80% SNP gate are shown in Fig. 2. Even at 80% SNP in the gate, the OTFT characteristics were good with very well defined linear and saturation regions, much lower OFF current and higher amplification as compared to OTFT with 100% SNP gate (Fig. S2c and d†). This indicates that even at composition of 80% SNP and 20% PEDOT:PSS, the composite electrode works effectively as gate electrode with good electronic conductivity.

**Fig. 3:**
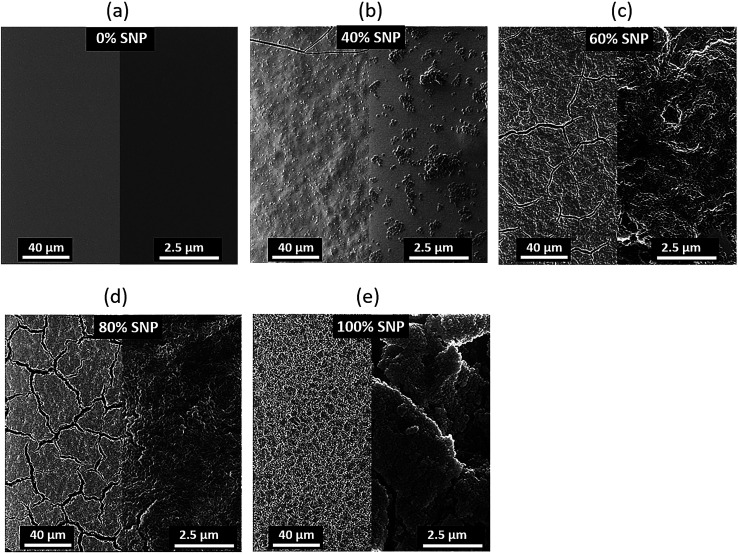
Helium ion microscope images of PEDOT:PSS : SNP composite gate electrodes with (a) 0%, (b) 40%, (c) 60%, (d) 80% and (e) 100% SNP.

To add to our understanding of the effect of composition of the PEDOT:PSS : SNP gate electrode on OTFT characteristics, we investigated the morphology of the composite gate electrodes. Helium ion microscope (HIM) images of the gate electrodes for five sets of gates are shown in Fig. 3. A reason for the major improvement in OTFT characteristics with composite gate electrodes even at only 20% PEDOT:PSS (80% SNP) as compared to OTFT with SNP only in the gate is revealed by their HIM images. As seen in Fig. 3e, at 100% SNP, the gate electrode looks uniform on a larger scale but on a smaller scale, the films have cracks and appears to show formation of clusters of SNP. With the addition of just 20% PEDOT:PSS, SNPs in the film are more connected and there are less cracks in the film (Fig. 3d). The cracks reduce further as the amount of PEDOT:PSS in the gate increases and at 60% of PEDOT:PSS, the PEDOT:PSS : SNP electrode does not display any cracks (Fig. 3b). As expected, at 40% SNP, the gate electrode is dominant with PEDOT:PSS and even though the film has no cracks, the SNPs do not form a continuous network. The role of PEDOT:PSS in the PEDOT:PSS : SNP gate electrodes is two-fold. Firstly, it improves the electronic conductivity of the gate (as compared to the gate with just SNP) and secondly, it acts as a binding agent for the SNPs. The improvement in the electronic conductivity of the gate electrode is easily seen from a simple current–voltage (*IV*) sweep of the gate electrode materials, provided in Fig. S3.† As expected, higher quantities of PEDOT:PSS leads to higher current for the same voltage. A 100% SNP film has much lower current and does not have a linear *IV* curve, which indicates that the SNP film by itself is a poor electronic conductor. For the composite electrodes, the *IV* curves are near linear for all compositions, even at very high SNP content (80%).

**Fig. 4:**
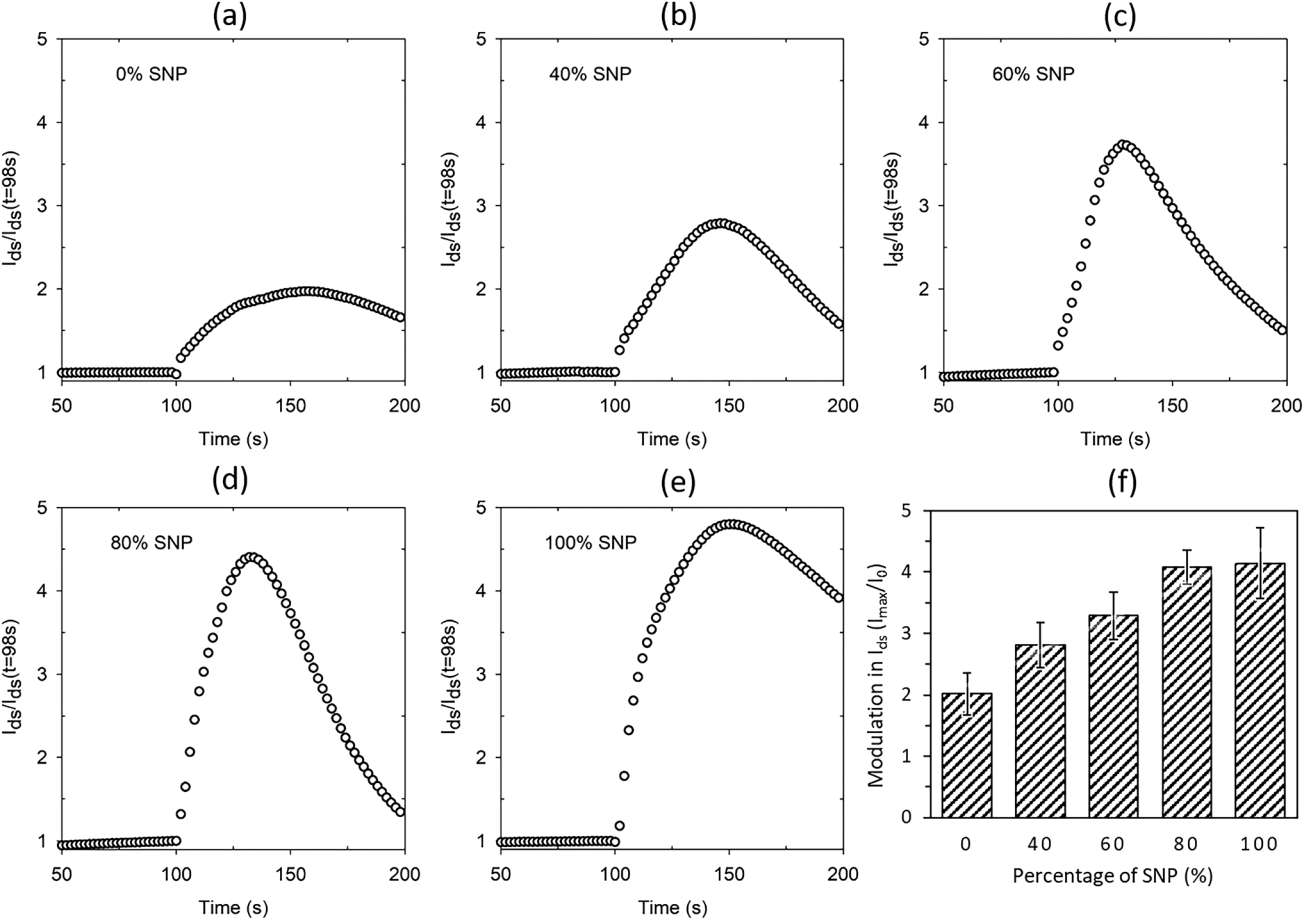
Normalised modulation in *I*_ds_ upon sensing protons for OTFTs with composite gate electrodes of (a) 0% SNP, (b) 40% SNP, (c) 60% SNP, (d) 80% SNP and (e) 100% SNP. (f) Average maximum modulation of *I*_ds_ for all the OTFTs with different gate composition.

The proton sensitivity of the five sets of OTFTs fabricated with gate electrode composition shown in Fig. 3 were tested using H_2_O_2_ in the same manner as described earlier. Typical normalized *I*_ds_ response curves are shown in Fig. 4a–e. The average maximum modulations are provided in Fig. 4f with standard deviation as error bars. As seen from the response curves and the average maximum modulation values, the modulation in *I*_ds_ increases with higher percentage of SNP in the gate electrode. Like before (Fig. S1a†), the modulation for the PEDOT:PSS gated OTFT is only a twofold modulation in *I*_ds_. However, the modulation increased to fourfold at 80% SNP in the gate. Although a higher percentage of PEDOT:PSS in the composite gate electrode leads to a more continuous gate film and better OTFT characteristics, a higher percentage of SNP leads to higher proton sensitivity. In Fig. 4, a 100% SNP gate electrode has the highest proton sensitivity, which is testament of the fact that SNP used in this study is one of the best solid-state proton conducting materials. Nevertheless, the increase in average proton sensitivity from 80% SNP in the gate to a 100% SNP gate is only a slight increase. This indicates that any percentage variation in quantity of SNP in the gate electrode between 80–100% will have very little difference in proton sensitivity.

Looking at the OTFT characteristics of the variations in composition of gate electrodes (Fig. 2 and S2†) and their proton sensitivity (Fig. 4), it may be concluded a composite gate PEDOT:PSS : SNP with 20 : 80 (v/v) is the best composition for the gate. In a win–win situation, the OTFT characteristics of this gate are very close to that of a PEDOT:PSS gated OTFT and also has proton sensitivity almost as high as the OTFT with 100% SNP gate. In Fig. 4, for all the gate compositions studied, the modulation in *I*_ds_ reduces after a maximum modulation as the gate electrode starts delaminating due to the analyte (H_2_O_2_), which contains water. Both PEDOT:PSS and SNPs solutions are water based and prolonged exposure to H_2_O_2_, which comes as a dispersion in water, delaminates the film. To make PEDOT:PSS films water stable, cross-linking of PEDOT:PSS is a widely used technique in OECTs.^18^ A similar strategy for the composite gate electrodes in this study is likely to lead to further robustness and all solid-state proton sensitive OTFTs that are stable in aqueous environments.

## 4. Conclusion

In summary, we have investigated the proton sensitivity of an all solid-state, low voltage operating OTFT with PEDOT:PSS, which is a mixed ionic/electronic conductor, as the gate electrode. We improved the proton sensitivity of the OTFT by mixing PEDOT:PSS with SNP and forming a composite gate electrode. The OTFTs with composite gate electrodes had OTFT characteristics very close to that of an OTFT with only PEDOT:PSS as the gate. PEDOT:PSS gated OTFTs showed a twofold modulation in *I*_ds_ upon sensing protons, whereas a composite gate electrode of 80% SNP showed a fourfold modulation in *I*_ds_. Our results provide new strategies for future directions for biomedical and bioelectronics sensors and transducers. Our OTFTs are directly applicable to biosensors where oxidase enzymes are used as the recognition element since oxidase enzymatic reactions produce H_2_O_2_ as a by-product. Ionic/protonic to electronic transducers are key to development of bioelectronic interfaces and our work serves as an important milestone towards achieving solution processable, all solid-state and low voltage operating transducers for such applications.

## Conflicts of interest

There are no conflict of interest to declare.

## Supplementary Material

RA-009-C9RA00148D-s001
