# Supplementary material for: A highly porous and conductive composite gate electrode for OTFT sensors
Source: RSC Adv. 2019 Mar 4;9(13):7278–84. doi: 10.1039/c9ra00148d (PMC9061173; doi:10.1039/c9ra00148d)
Supplement: RA-009-C9RA00148D-s001 [file RA-009-C9RA00148D-s001.pdf]

Electronic Supplementary Material (ESI) for RSC Advances.
This journal is © The Royal Society of Chemistry 2019

Supplementary information

A highly porous and conductive composite gate electrode for OTFT sensors

Soniya D. Yambem,[ab] Samantha Burns,[a] Joshua N. Arthur,[ab] Jana Timm,[c] Maria A. Woodruff,[ab] Ajay K. Pandey[bd] and Roland Marschall[c]*

[a]School of Chemistry Physics and Mechanical Engineering, Science and Engineering Faculty, Queensland University of Technology (QUT), Brisbane QLD 4000, Australia
[b]Institute of Health and Biomedical Innovation, Queensland University of Technology (QUT), Kelvin Grove, Queensland 4059, Australia
[c]Department of Chemistry, University of Bayreuth, Bayreuth 95447, Germany
[d]School of Electrical Engineering and Computer Science, Science and Engineering Faculty, Queensland University of Technology (QUT), Brisbane QLD 4000, Australia

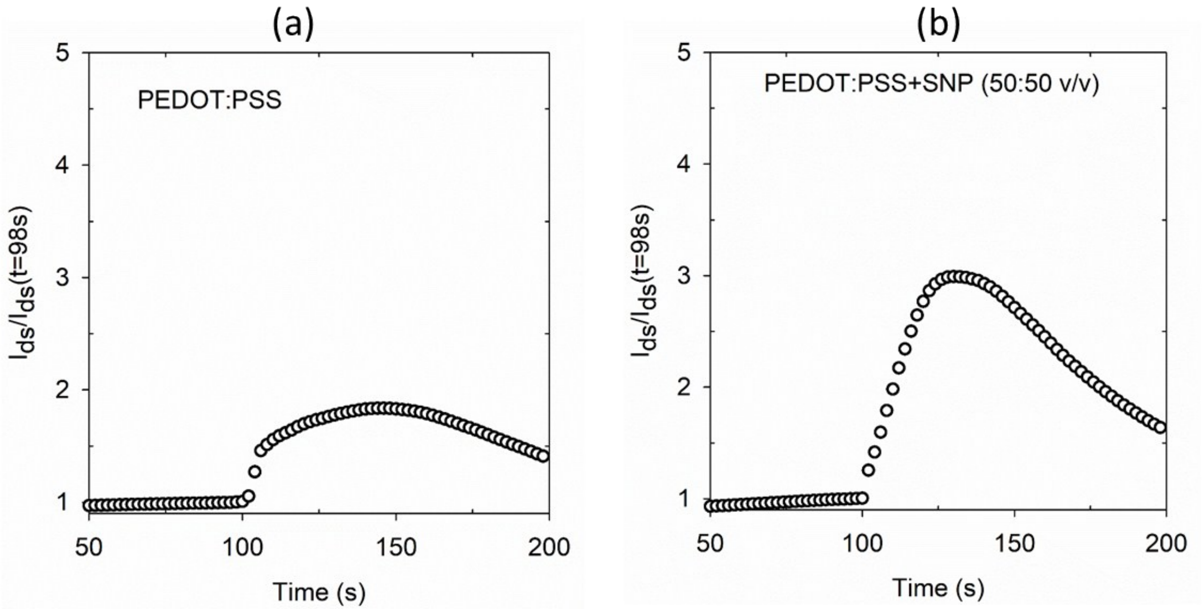

Figure S1. Normalised modulation in I_{ds} upon sensing of protons for (a) OTFT with PEDOT:PSS gate and (b) PEDOT:PSS:SNP (50:50, v/v) gate.

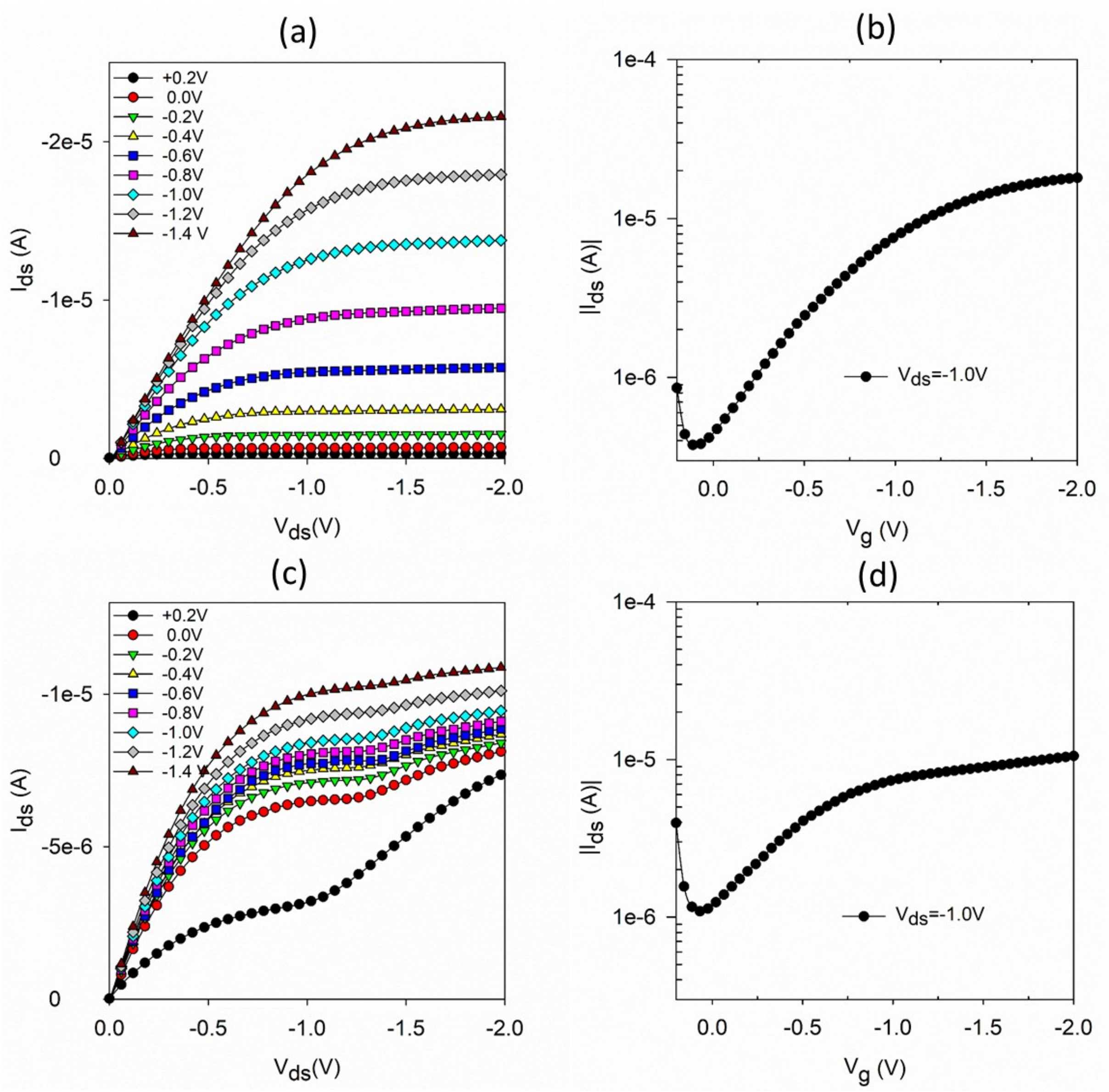

Figure S2. (a) Output and (b) transfer characteristics of OTFT with PEDOT:PSS gate (0% SNP). (c) Output and (d) transfer characteristics of OTFT with SNP gate (100% SNP).

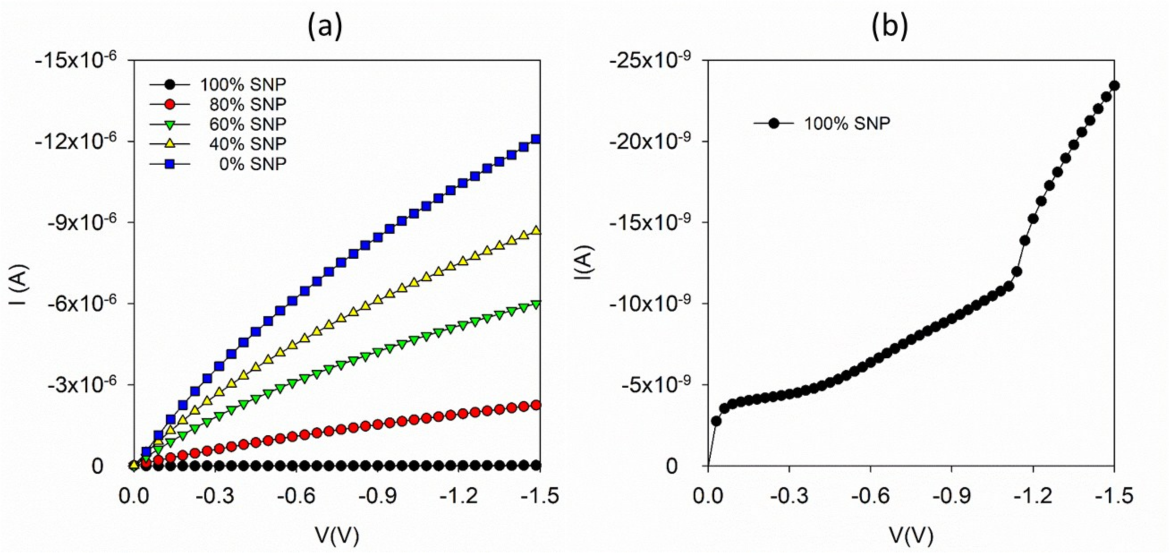

Figure S3. Current-voltage sweeps of gates electrode materials. The films had the same dimensions. They were deposited on ITO contact pads.
